# TGF-β1/CD105 signaling controls vascular network formation within growth factor sequestering hyaluronic acid hydrogels

**DOI:** 10.1371/journal.pone.0194679

**Published:** 2018-03-22

**Authors:** Shane Browne, Amit K. Jha, Kurosh Ameri, Sivan G. Marcus, Yerem Yeghiazarians, Kevin E. Healy

**Affiliations:** 1 Departments of Bioengineering and Materials Science and Engineering, University of California, Berkeley, CA, United States of America; 2 Centre for Research in Medical Devices (CÚRAM), National University of Ireland Galway, Galway, Ireland; 3 Department of Medicine, University of California, San Francisco, CA, United States of America; 4 Eli and Edythe Broad Center of Regeneration Medicine and Stem Cell Research, University of California, San Francisco, CA, United States of America; 5 Cardiovascular Research Institute, University of California, San Francisco, CA, United States of America; Universita degli Studi di Bari Aldo Moro, ITALY

## Abstract

Cell-based strategies for the treatment of ischemic diseases are at the forefront of tissue engineering and regenerative medicine. Cell therapies purportedly can play a key role in the neovascularization of ischemic tissue; however, low survival and poor cell engraftment with the host vasculature following implantation limits their potential to treat ischemic diseases. To overcome these limitations, we previously developed a growth factor sequestering hyaluronic acid (HyA)-based hydrogel that enhanced transplanted mouse cardiosphere-derived cell survival and formation of vasculature that anastomosed with host vessels. In this work, we examined the mechanism by which HyA hydrogels presenting transforming growth factor beta-1 (TGF-β1) promoted proliferation of more clinically relevant human cardiosphere-derived cells (hCDC), and their formation of vascular-like networks *in vitro*. We observed hCDC proliferation and enhanced formation of vascular-like networks occurred in the presence of TGF-β1. Furthermore, production of nitric oxide (NO), VEGF, and a host of angiogenic factors were increased in the presence of TGF-β1. This response was dependent on the co-activity of CD105 (Endoglin) with the TGF-βR2 receptor, demonstrating its role in the process of angiogenic differentiation and vascular organization of hCDC. These results demonstrated that hCDC form vascular-like networks *in vitro*, and that the induction of vascular networks by hCDC within growth factor sequestering HyA hydrogels was mediated by TGF-β1/CD105 signaling.

## Introduction

Cell therapies hold great promise for the treatment of a range of pathological conditions.[1–3] However, poor cell survival following transplantation has limited the effectiveness of cell therapies, and delayed their clinical translation. This issue is related to the harsh environments into which stem cells are typically delivered. In particular, ischemia leads to poor cell survival and engraftment within the diseased tissue.

Biomaterial systems have been proposed as a means by which to promote angiogenesis[4–6] and enhance the survival of transplanted stem cells to treat ischemia[7–9]. By recapitulating key aspects of the extracellular matrix (ECM) such as mechanical properties, ligand interactions, and cell-mediated degradation/remodeling, biomaterial systems can enhance retention and survival of transplanted cells, in addition to protecting the cells from the harsh ischemic microenvironment including radical oxygen species (ROS).[10] These biomaterials systems fall broadly into two categories, either natural, or synthetic. Major components of the native ECM have been to the fore in this regard, particularly collagen,[11–13] and hyaluronic acid (HyA).[14–16] Other biopolymers such as fibrin[17], alginate[18] and chitosan[19] have also been studied, along with Matrigel™[20], a heterogeneous ECM derived from mouse sarcoma cells, and platelet-lysate.[21] However, batch-to-batch variation, along with a lack of control over a range of matrix parameters such as mechanical properties and degradation, has limited the enthusiasm for these natural biopolymers. In contrast, synthetic and semi-synthetic materials are reproducible and scalable, amenable to chemical modification and can be precisely tuned for specific properties.[22,23] Examples of synthetic materials include the self-assembling RADA16 peptide[24] and NIPAAM-based polymers[25], which have been tested in pre-clinical models of ischemia and enhanced cell retention. However, synthetic materials lack inherent bioactivity and therefore typically underperform compared to semisynthetic matrices (sECM), where natural biopolymers are modified to impart instructive bioactivity. Of all the sECMs being developed, those employing HyA appear to have the most potential.[26] HyA matrices promote the survival of a range of stem cells including MSCs,[27,28] neural progenitor cells(NPCs)[29] and hCDC.[30,31] In the context of angiogenesis, adipose-derived stem cells (ADSCs) in a functionalized HyA matrix promoted angiogenesis in an ischemic model, resulting in reduced tissue fibrosis.[32] HyA matrices have also enhanced wound healing by promoting vascularization using transplanted endothelial progenitors.[33]

Here we propose matrix-assisted cell transplantation (MACT) using a semi-synthetic acrylate-modified HyA hydrogel as a means to promote cell survival and engraftment, and subsequent angiogenesis and tissue regeneration. We employed a HyA hydrogel decorated with a bsp-RGD(15) peptide sequence for cell adhesion,[34–36] high molecular weight heparin to sequester growth factors,[37] and an MMP-cleavable peptide crosslinker to allow for cell-mediated matrix-remodeling.[38,39] This HyA system allows for independent control over key parameters such as adhesion ligand density, growth factor sequestration and presentation, mechanical properties, and degradation rates, in contrast with biological materials that do not allow for such specifically tunable systems. Previously we demonstrated that these HyA matrices directed the formation of vascular networks by encapsulated mouse Sca1^+^/CD45^-^ cardiac progenitor cells when TGF-β1 was exogenously sequestered within the matrix.[34,38]

In this study, we used these growth factor sequestering HyA matrices to interrogate the role of TGF-β1 signaling, via CD105 (Endoglin) in vascular differentiation and network formation using a more clinically relevant population of human cardiosphere derived cells (hCDC). In contrast with the anti-angiogenic effects of TGF-βR1, signaling through TGF-βR2 and the CD105 co-receptor plays an important role in endothelial cell proliferation and migration. It has been shown that CD105 enhances angiogenesis by increasing signaling through ALK1 and reducing signaling through ALK5.[40] The CD105 receptor is highly expressed in hCDC,[41,42] and has been shown necessary to stimulate pro-angiogenic paracrine support by murine CDCs.[43] Therefore we investigated whether CD105 coordinates with the TGF-βR2 receptor and TGF-β1 presented to hCDC in our HyA hydrogels to drive vascular network formation *in vitro*.

## Results

### Gel formation and cell encapsulation

**Fig 1:**
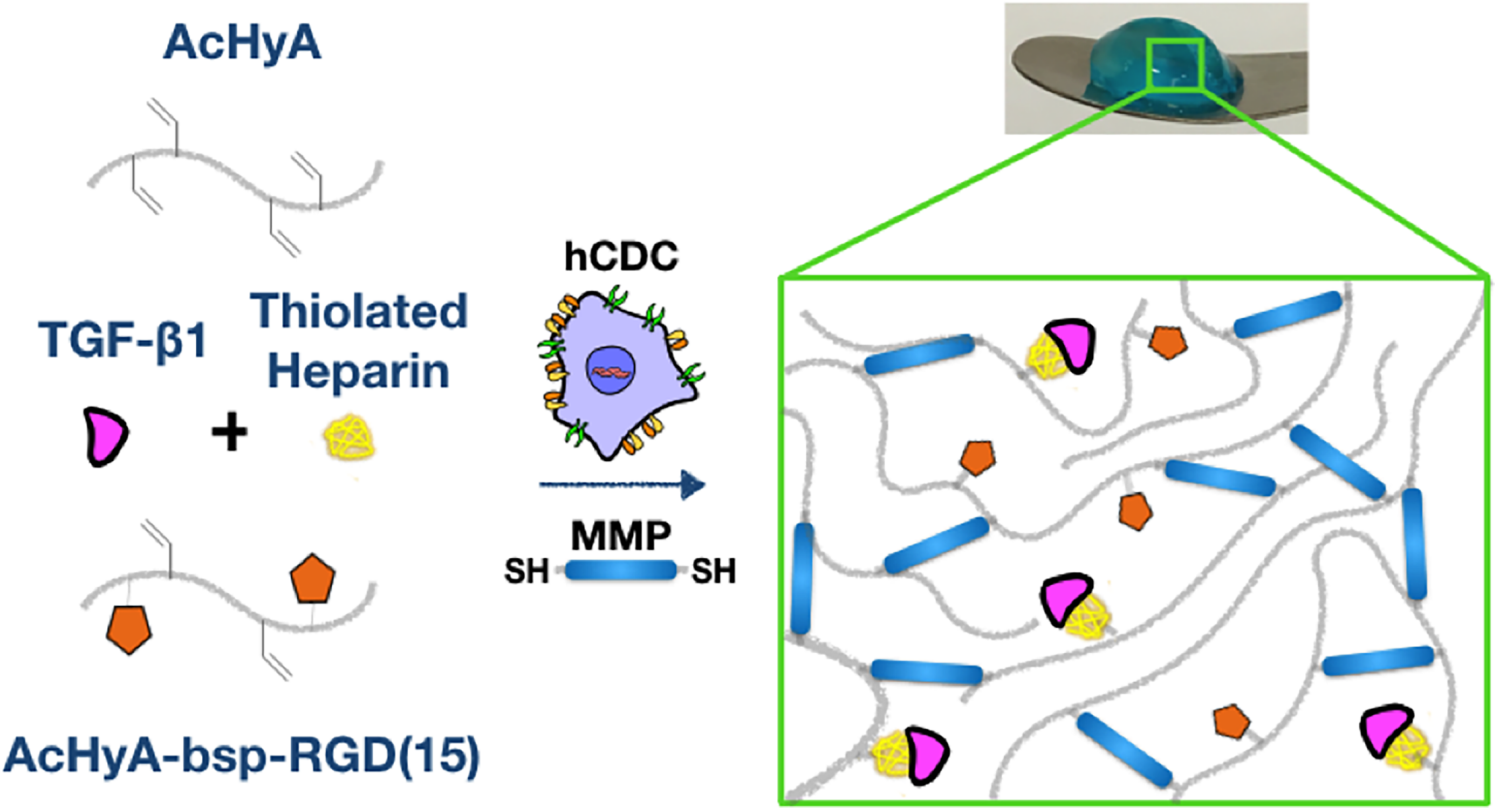
Schematic of encapsulation of human cardiosphere-derived cells (hCDC) within growth factor-sequestering hydrogels. Hydrogel formation occurs though Michael-type addition of cysteine-terminated MMP peptides to the functionalized hyaluronic acid macromers (AcHyA), which crosslinks the hydrogel. This reaction occurs in the presence of thiolated heparin, TGF-β1 and human hCDC, which allows for cell encapsulation within the hydrogel, and additionally the grafting of heparin to the hydrogel, which then binds TGF-B1 via its natural heparin binding domain. Gel is stained blue for clarity.

**Fig 2:**
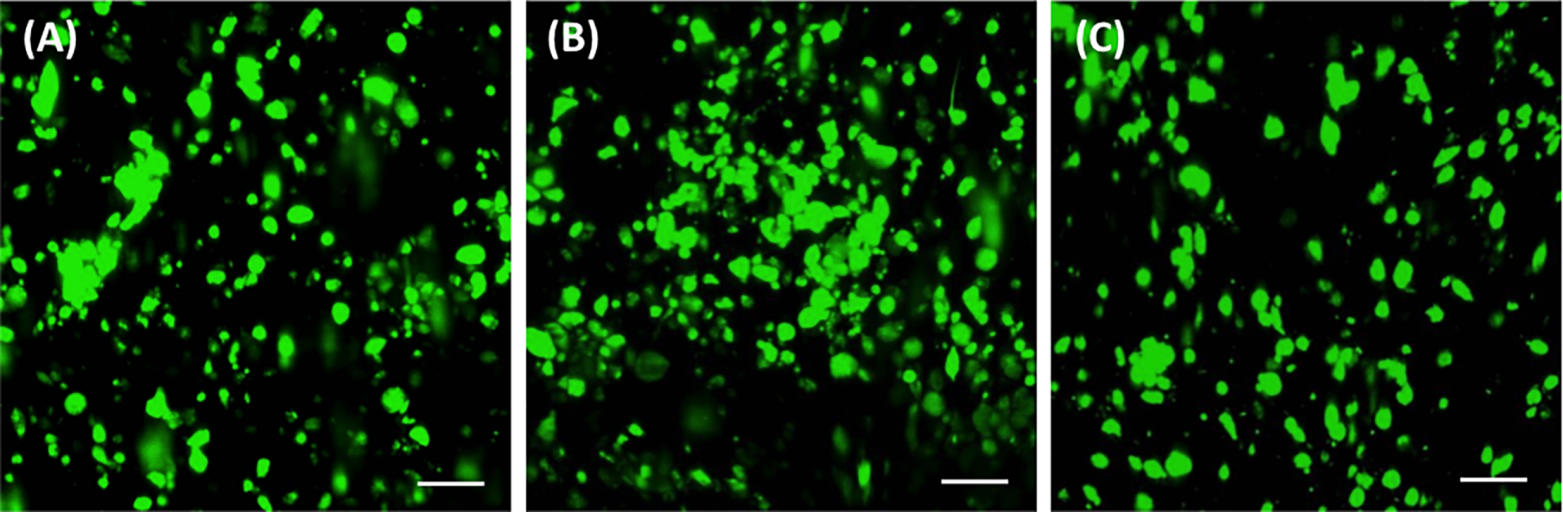
Survival of hCDC 24 hours after encapsulation in HyA hydrogels. Live/Dead staining of matrices (**A**) HyA, (**B**) HyA-TGF-β1 and (**C**) HyA-TGF-β1/CD105 Ab blocking. Scale Bar = 100 μm.

**Fig 3:**
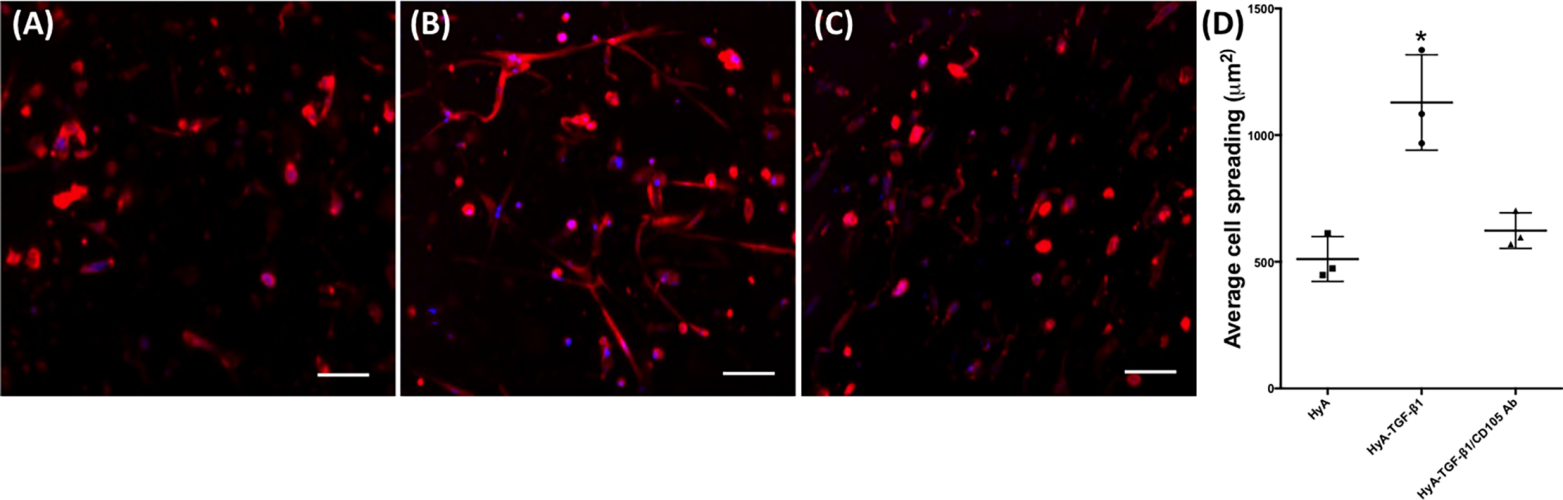
Spreading of hCDC to HyA matrices after 3 days. Actin stain of hCDC encapsulated in (**A**) HyA, (**B**) HyA-TGF-β1 and (**C**) HyA-TGF-β1/CD105 Ab blocking. (**D**) Quantification of cell area shows that TGF-β1 significantly enhances the spreading of hCDC, but that this effect can be abrogated by pre-incubation with CD105 antibody. Data represents mean ± SD (n = 3). Analysis performed using one-way ANOVA, p < 0.05. * denotes statistical significance. Scale Bar = 100 μm.

Gelation of growth factor-presenting HyA hydrogels occurred through Micheal-type addition of bis-cysteine MMP-cleavable peptides to HyA macromers, AcHyA and AcHyA-bsp-RGD(15), and soluble thiolated heparin (**Fig 1A**). The hCDC survived this encapsulation process with no significant sign of cell death in any of the groups assessed after 24 hours (**Fig 2**). The presence of TGF-β1 in the matrix greatly enhanced the spreading of hCDC compared to HyA matrices without TGF-β1, as observed with actin staining. (**Fig 3A and 3B**). This effect was abrogated by pre-incubation of the hCDC with antibody blocking CD105, emphasizing the role of TGF-β1/CD105 interactions (**Fig 3C**). Quantification of cell spreading (**Fig 3D**) revealed a significant difference in cell area between the HyA hydrogel group (511 ± 89 μm^2^), and the HyA + TGF-β1 group (1129 ± 188 μm^2^), and those where TGF-β1/CD105 activity was repressed by blocking antibody (623 ± 70 μm^2^).

### Cell proliferation with HyA hydrogels

**Fig 4:**
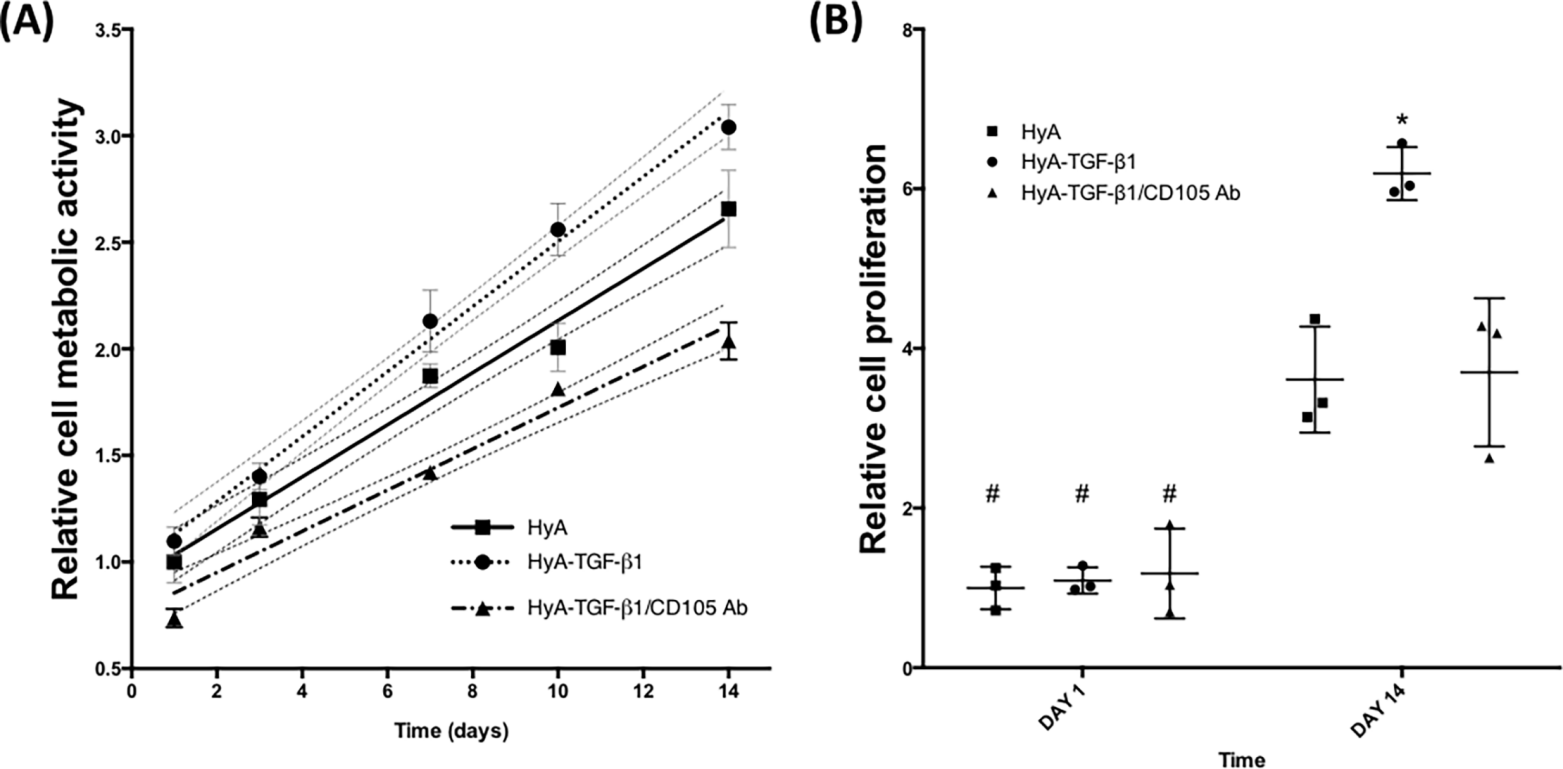
hCDC metabolic activity and proliferation over 14 days. (**A**) Proliferation curves (showing 95% CI) of hCDC encapsulated within HyA matrix demonstrates an increase due to TGF-β1. (**B**) DNA content of HyA matrices from 1 to 14 days shows a significant effect of TGF-β1, which is negated by pre-incubation with CD105 antibody. Data represents mean ± SD (n = 3). Analysis performed using one-way ANOVA, p < 0.05. * denotes statistical significance compared with other groups at 14 days, # denotes statistical significance versus same group at 14 days.

The metabolic activity, representative of proliferation, of hCDC following incorporation of TGF-β1 in the HyA system is shown in **Fig 4A**. After 14 days, there was an increase in cell number in TGF-β1 containing HyA hydrogels compared with either of the other groups. The HyA matrix without TGF-β1 showed the least increase in proliferation activity over 14 days, with blocking TGF-β1 activity by CD105 antibody reducing metabolic activity compared with the TGF-β1 alone treatment. Linear regression revealed that each group had a significantly different slope of increasing metabolic activity over the 14 day period, with the greatest slope in the TGF-β1 treated group (0.153 ± 0.01) compared with the untreated group (0.122 ± 0.01), and the TGF-β1/CD105 antibody blocking group (0.097 ± 0.01)

A similar effect was observed in terms of DNA content of the hydrogels. Groups were compared at 1 and 14 days following encapsulation. On day one, there was no difference in the DNA content of any of the groups regardless of the presence of TGF-β1 or blocking CD105 activity (**Fig 4B**). However, by 14 days, there was a significant increase in DNA content in all of the groups, compared to day 1. The greatest increase was observed following treatment with TGF-β1, and DNA content of this group was significantly greater than the other two groups at 14 days. There was no significant difference between the pre-treatment with CD105 antibody and HyA hydrogel group without TGF-β1 groups.

### TGF-β1 –induced endothelial differentiation of encapsulated hCDC

**Fig 5:**
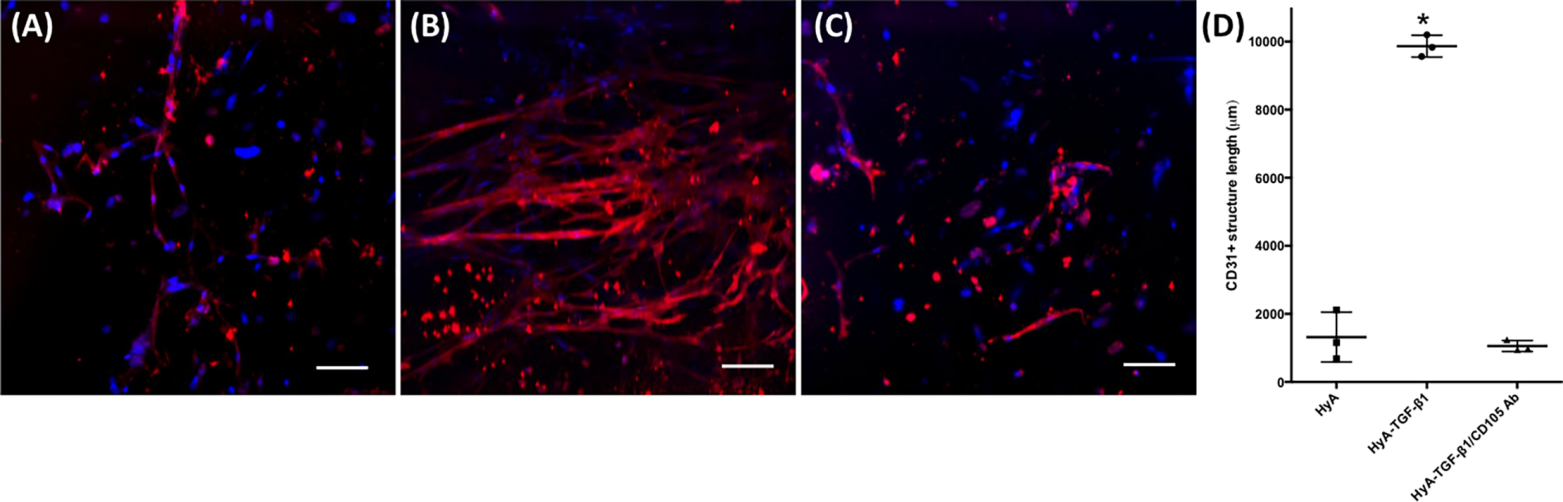
Vascular network formation in HyA matrix. Expression of CD31 after 14 days of encapsulation of hCDC in (**A**) HyA (**B**) HyA-TGF-β1 and (**C**) HyA-TGF-β1/CD105 Ab. (**D**) Quantification of the length of CD31+ structures reveals a significant effect of TGF-β1 on the formation of CD31+ networks. Data represents mean ± SD (n = 3). Analysis performed using one-way ANOVA, p < 0.05. * denotes statistical significance. Scale Bar = 100 μm.

There was a significant difference in CD31 expression between groups after 14 days in culture. A vast interconnected network of CD31 expressing cells formed throughout the HyA hydrogel when TGF-β1 was present (**Fig 5A**). In contrast, only sparse networks were observed in hydrogels without TGF-β1 and those pre-incubated with CD105 antibody prior to their encapsulation within a TGF-β1 containing HyA hydrogel (**Fig 5B and 5C**). Quantification of the length of CD31 positive structures within each group demonstrated a clear difference (**Fig 5D**), with the HyA-TGF-β1 group having a much greater average length of CD31+ structures (9870 ± 320 μm). In comparison, the HyA group (1320 ± 730μm) or the HyA-TGF-β1/CD105-blocking group (1060 ± 150 μm) had greatly reduced length of CD31+ structures.

**Fig 6:**
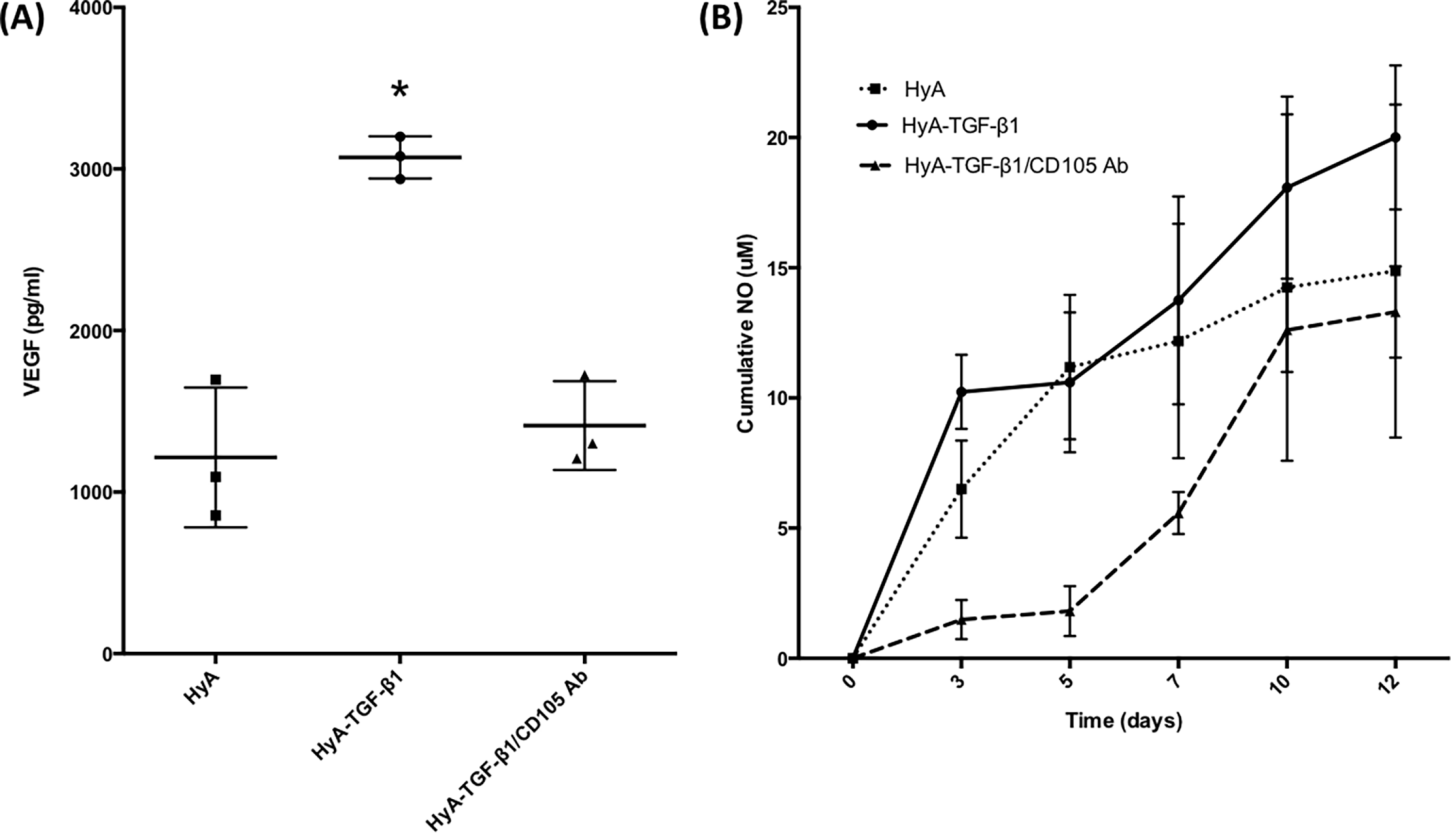
VEGF and NO expression in hCDC. (**A**) TGF-β1 enhances the production of VEGF by hCDC within HyA matrices, an effect which can be reduced by blocking the CD105 receptor. (**B**) Cumulative expression of NO over 14 days in hCDC encapsulated in HyA matrices reveals the effect of TGF-β1 on NO production. Data represents mean ± SD (n = 3). Analysis performed using one-way ANOVA, p < 0.05. * denotes statistical significance.

The presence of TGF-β1 enhanced the production and subsequent sequestration of endogenously synthesized VEGF within the matrix. **Fig 6A** shows that there was a significant increase in the levels of sequestered VEGF with the inclusion of TGF-β1 within the matrix (3070 ± 130 pg/mL) compared with the other groups. In fact, in comparison with either HyA matrix without the presence of TGF-β1 (1220 ± 430 pg/mL) or the hCDC treated with TGF-β1, but pre-incubated with CD105 blocking antibody (1410 ± 280 pg/mL) there was approximately a three-fold increase in VEGF levels. Increased levels of nitric oxide (NO) were also detected in the presence of TGF-β1 (**Fig 6B**). Over 14 days, the levels of NO increased in the HyA-TGF-β1 group, compared with the other groups. Particularly from days 8–14, the levels of NO increased dramatically in the HyA-TGF-β1 group (12.37 ± 1.25 μM to 26.34 ± 2.92 μM), compared with the others where a minimal increase was detected. The HyA group (8.06 ± 3.5 μM to 13.79 ± 0.54 μM) and the HyA-TGF-β1/CD105-blocking group (8.59 ± 3.72 μM to 11.22 ± 3.49 μM) were broadly similar during this time period.

### Paracrine mechanism of endothelial differentiation and network formation

**Fig 7:**
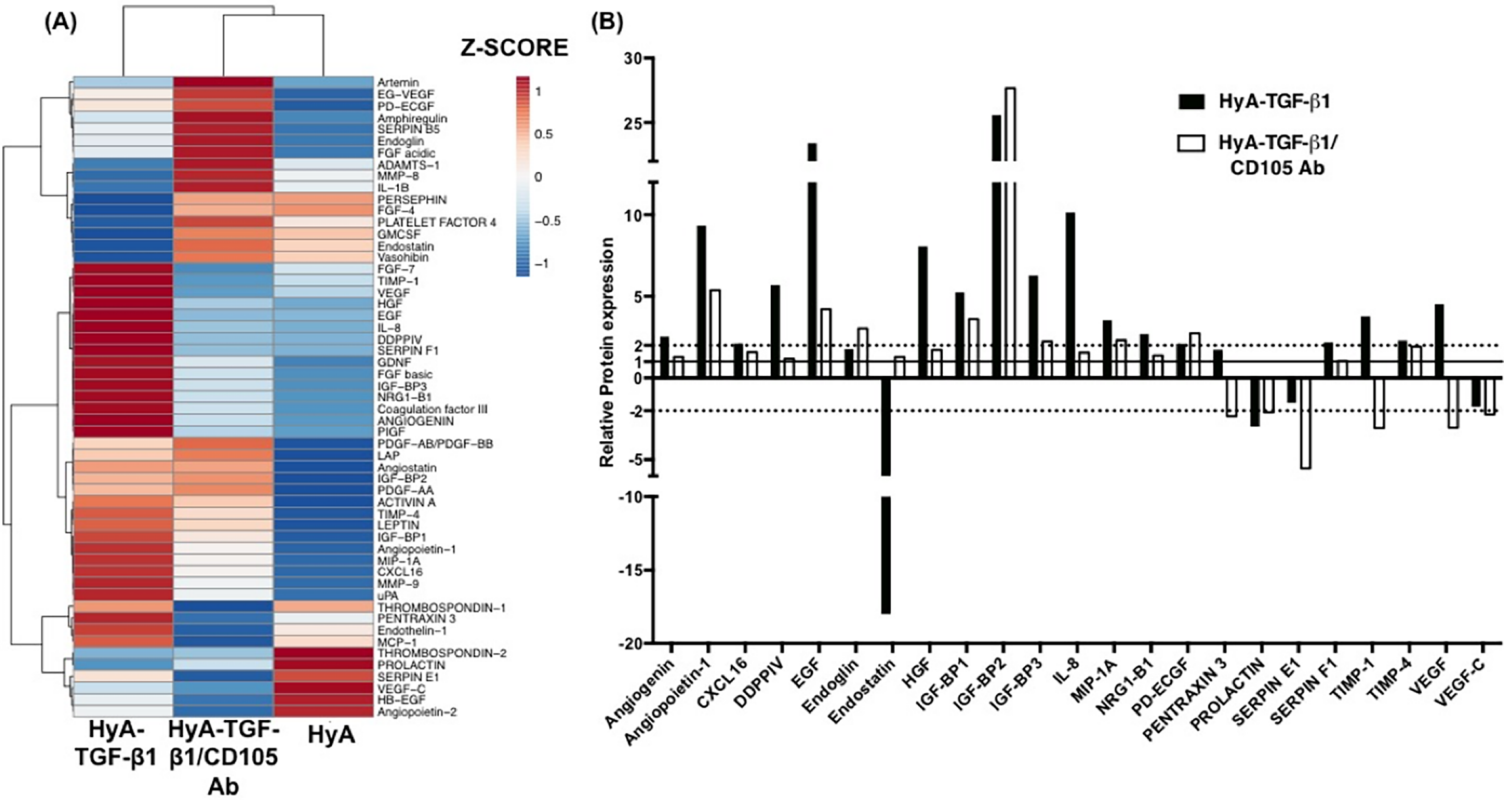
Angiogenic paracrine secretions of hCDC sequestered in HyA matrices at 14 days. (**A**) Heatmap dendrogram of z-score expression of a range of angiogenesis-related factors. (**B**) Relative protein expression of angiogenic factors that are up- or down-regulated in the HyA-TGF-β1 group compared to that group with blocked CD105 receptor. The incorporation of TGF-β1 within the HyA matrices stimulates the secretion of a range of pro-angiogenic factors within HyA matrices, and many of these factors are negatively regulated by blocking the CD105 receptor on hCDC.

To demonstrate the effect of TGF-β1/CD105 activity on the expression of angiogenic factors, angiogenesis-related factors that were endogenously synthesized by the hCDC and sequestered within the HyA hydrogel were compared as a heatmap dendrogram of z-scores (**Fig 7A**). A striking difference was observed between the HyA-TGF-β1 matrices and the other two groups (**Fig 7A**), with the blocking of CD105 activity dramatically changing the expression profile where clustering was observed in the expression of a number of pro-angiogenic factors. For example, known pro-angiogenic factors such as VEGF, HGF, EGF, IL-8, bFGF, Angiogenin and Angeopoetin-1 were upregulated with the addition of TGF- β1 to the matrix. There was also an increase in PDGF-AA, PDGF-AB/BB, and some matrix remodeling factors such as MMP-9 and TIMP-4. In contrast, these factors are mostly downregulated in either the non-TGF-β1 treated group or the TGF-β1/CD105 blocking antibody group. Hierarchical clustering revealed that the HyA group and the HyA-TGF-β1/CD105 Ab blocking group were most similar, while the TGF-β1 treated group was different when compared to the aforementioned groups.

To observe the effect of either TGF-β1 or of blocking its activity through the CD105 receptor, the relative expression of each factor was normalized to the non-TGF-β1 treated group. A cut-off of up or down-regulation of +2 or -2 was applied, and the relative expression of all the proteins meeting these criteria plotted in **Fig 7B**. Factors which were highly upregulated in the presence of TGF-β1 include CXCL16, EGF, HGF, IGF-BP1/3, IL-8, TIMP-1 and VEGF. The endogenous angiogenesis inhibitor Endostatin was downregulated in the presence of TGF-β1, while the presence of the CD105 blocking antibody negated this effect of TGF-β1 on Endostatin. VEGF expression was also downregulated in the presence of the CD105 blocking antibody, in comparison with the non-TGF-β1 treated group, and particularly the TGF-β1 group where it was significantly upregulated.

## Discussion

Vascularization is not only a key process for the survival of transplanted cells, but also as a potential treatment for ischemic tissues. Our recent work has focused on the development of semi-synthetic HyA-based hydrogel systems for enhanced survival of transplanted angiogenic cells. HyA is an ideal candidate material for matrix assisted cell transplantation given its capacity for modification and biofunctionalization. We have identified hydrogel parameters, in terms of growth factor concentration and sequestration[37] and matrix degradation,[38] to promote the formation of vascular networks. In this study, we examined the mechanism by which TGF-β1-stimulated vascular differentiation and network formation of hCDC within these HyA hydrogels.

Vascular network formation of hCDC within our HyA matrices was dependent on TGF-β1 signaling through the CD105 receptor. The short-term survival of hCDC within the HyA hydrogels was independent of the presence of TGF-β1. However, the adhesion and spreading of hCDC was dependent on the activity of TGF-β1, which was negated by pre-incubation of hCDC with a CD105 blocking antibody. Similar observations were made for metabolic activity and proliferation, where TGF-β1 activity was required for optimal behavior, suggesting TGF-β1 signaling through the CD105 receptor was a necessity. We suggest these observations are due to both the reduction in TGF-β1 signaling through the CD105 receptor, and the interaction between the CD105 receptor and αV and α5 integrins involved in cell adhesion.[44]

Pre-clinical studies involving transplantation of hCDCs have classically reported enhanced vascularization in the infarcted myocardium.[45,46] Hence, *in situ* differentiation to endothelial cells and the formation of vascular networks within HyA matrices were key aims of this study. HyA matrices containing hCDC were stained for the endothelial cell surface marker CD31, to examine both the relative level of endothelial cell differentiation, and the formation of vascular networks. In the presence of TGF-β1, dense and complex networks of CD31+ cells formed, whereas sparse cell dispersions were formed without TGF-β1 or where CD105 was blocked.

The expression of NO and VEGF were modulated by TGF-β1-CD105 signaling. Nitric oxide (NO), a product of endothelial nitric oxide synthase (eNOS) is an important mediator in terms of angiogenesis and vascular tone,[47,48] and increased eNOS activity has been shown to increase angiogenesis.[49] Furthermore, eNOS activity is a characteristic of endothelial cells, and thus increased levels of NO are indicative of endothelial cell activity. The hCDC encapsulated within TGF-β1 containing matrices exhibited enhanced NO and VEGF production compared with all other groups. VEGF is a key driver of angiogenesis, proliferation and migration of endothelial cells,[50,51] and thus increased VEGF levels are consistent with the formation of CD31+ networks in the TGF-β1 containing matrices. Furthermore, studies link NO and VEGF expression, with various reports indicating a direct relationship between the two.[52,53] Thus, the consistency we observe in terms of NO and VEGF expression between groups, where higher levels of NO are in line with higher VEGF expression and vice-versa is in line with previous observations.

To better understand how CD105/TGF-β1 signaling shifted hCDC to an angiogenic phenotype, we explored the angiogenic proteins endogenously secreted by encapsulated hCDC and sequestered within the HyA matrix. In the TGF-β1 containing HyA matrices, there was a significant upregulation of a range of pro-angiogenic factors, particularly factors typically associated with angiogenesis such as Angiogenin, Angiopoietin-1, EGF, HGF, IL-8 and VEGF. A number of these factors have previously been shown to be expressed by hCDC in 2D culture, where the paracrine secretions of hCDC were shown to be superior to a number of other stem cell populations.[54] In other 3D systems, the expression of angiogenic factors such as Angiogenin, IGF-1, IL-6, SDF-1α and VEGF has previously been reported.[55,56] An increase in TIMP-1 and MMP-8 expression also indicates the involvement and regulation of MMPs in the processes of endothelial cell differentiation of hCDC and subsequent vascular formation, while the upregulation of insulin-like growth factor (IGF)-binding proteins, particularly IGF-BP3, indicates a potential role for IGF. Interestingly, endostatin, an endogenous angiogenesis inhibitor, was downregulated by hCDC encapsulated in TGF-β1 containing HyA matrices, which indicates that the hCDC have shifted to an angiogenic phenotype. One limitation to our observations is that the expression was analyzed at 14 days, while angiogenesis is a temporal process involving a number of phases, with phases driven by different factors. Thus, future studies need to focus on detailed temporal analysis of the key factors involved in this process. However, what this work proves is that TGF-β1 stimulates an increase in a range of pro-angiogenic factors secreted by hCDC, which can be negated by the use of a CD105 blocking antibody. Thus, the role of TGF-β1 signaling through CD105 is clear with regard to the stimulation of the increased production and secretion of a range of pro-angiogenic factors by hCDC.

HyA is a polysaccharide which has been widely used in the field of regenerative medicine, owing to its ease of modification, bioactivity and biodegradation.[57] Specifically, HyA has been routinely used as the base polymer to design sECM for cell encapsulation.[15,28,58–60] HyA has also been associated with inflammation and angiogenesis in a number of disease states, notably cancer.[61,62] These effects have been correlated with molecular weight, with lower molecular weight HyA being pro-inflammatory and pro-angiogenic and higher molecular weight HyA having the opposite effects. Specifically, degradation products of 3–16 disaccharides have been shown to have pro-angiogenic effects *in vitro* and *in vivo*.[63,64] However, in the context of our HyA system, we observed no signs that 500 kDa HyA in its crosslinked form supports the differentiation of hCDC to an angiogenic phenotype without the addition of TGF-β1.

**Fig 8:**
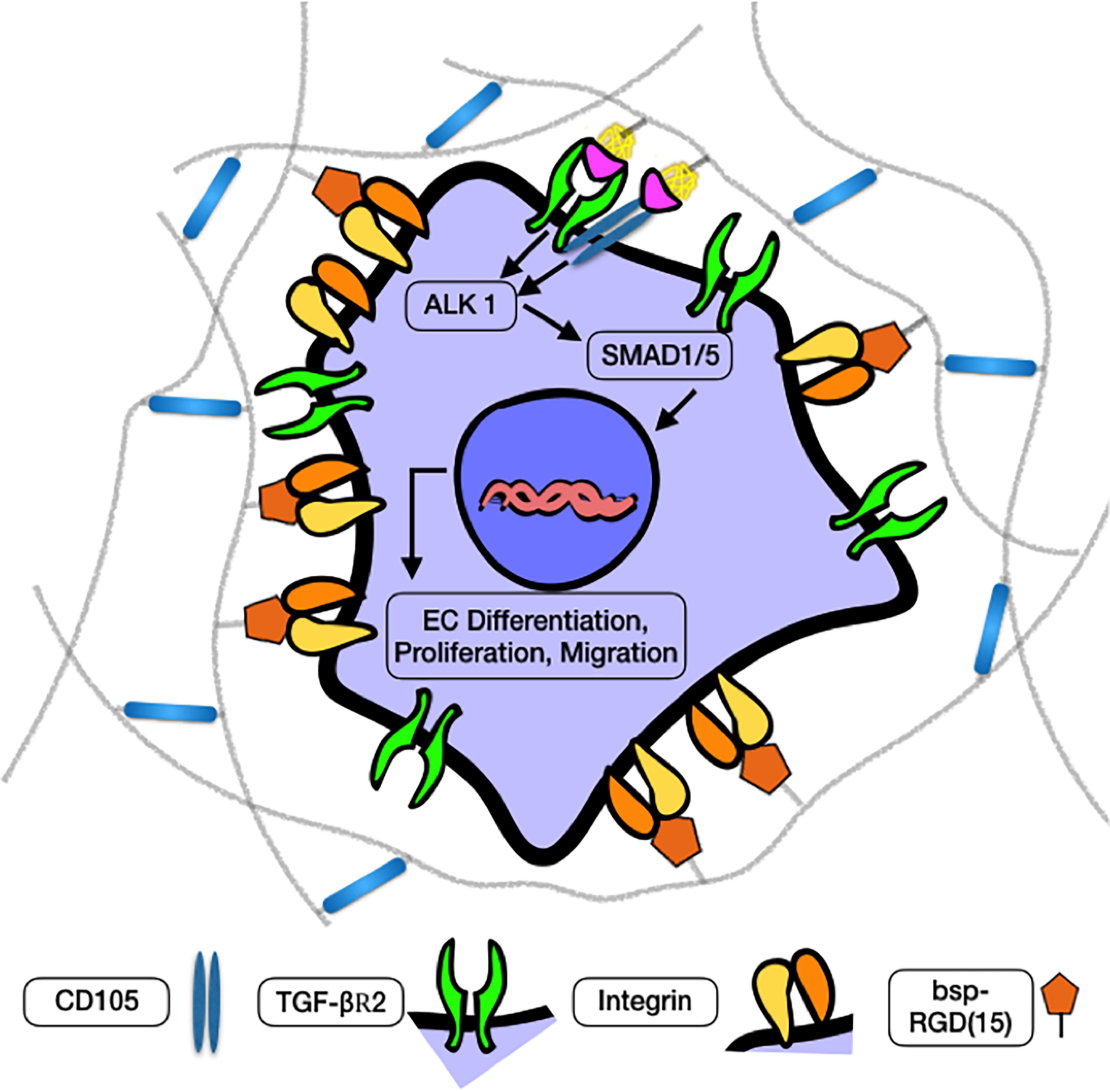
Schematic depicting the mechanism of vascular differentiation and network formation by encapsulated hCDC. Presentation of heparin-sequestered TGF-β1 to the TGF-βR2 and the CD105 co-receptor activates the ALK 1 and SMAD 1/5 pathways, resulting in increased angiogenic activity of encapsulated hCDC.

In relation to the mechanism of angiogenesis, CD105 has been implicated in controlling the effect of TGF-β1, by countering its inhibitory effect on proliferation, migration and tube formation in endothelial cells.[65] The use of CD105 blocking antibodies has previously been shown to suppress the growth of endothelial cells *in vitro*, with the addition of TGF-β1 synergistically suppressing cell proliferation.[66] Thus, by blocking CD105, the inhibitory effects of TGF-β1, rather than stimulatory effects, on endothelial cells prevail. In a different context, we see a similar effect, in that blocking CD105 impedes a range of pro-angiogenic effects of TGF-β1 when exposed to hCDC in our 3D HyA matrix. These results indicate that the induction of an angiogenic hCDC phenotype by TGF-β1 containing HyA matrices is dependent on signaling through the CD105 receptor (**Fig 8**). In fact, TGF-β1 signaling via the CD105 receptor has been shown to shown to play a role in endothelial cell proliferation and activin receptor-like kinase 1 (ALK1) signaling.[40,67] TGF-β1 signaling through the CD105 receptor has been shown to activate Smad1 and Smad5, leading to enhanced proliferation, migration and organization of endothelial cells.[68,69] CD105 knockout mice fail to survive beyond 10.5 days poscoitum, failing to form mature blood vessels in the yolk sac.[70] Furthermore, deformities in cardiac development are also detected in CD105 knockout mice.[70] Collectively these data indicate a critical role for CD105 and TGF-β1 signaling in promoting vascular network formation by hCDC in HyA hydrogels. Interestingly, CD105 expression is also increased in hypoxic conditions.[71] Thus, our system may mimic and/or augment signaling needed during ischemia (to induce angiogenesis and also prevent death of transplanted cells), which will be desirable for in vivo applications.

## Conclusions

Semi-synthetic HyA hydrogels facilitate the differentiation of hCDC into endothelial cells, and the formation of CD31-expressing networks. This occurs through the presentation of TGF-β1 and signaling through CD105. TGF-β1 signaling via the CD105 receptor controls a myriad of cell functions leading up to vascular network formation, including cell spreading and proliferation, production of key angiogenic factors, and endothelial differentiation. The combination of hCDC and TGF-β1-presenting growth factor sequestering HyA matrix has potential as an injectable therapeutic for treating ischemic injury.

## Methods

### Materials

Hyaluronic acid (HyA, sodium salt, 500 kDa) was purchased from Lifecore Biomedical (Chaska, MN). Adipic dihydrazide (ADH), 1-ethyl-3-[3-(dimethylamino)propyl] carbodiimide (EDC), sodium hydroxide (NaOH), hydrochloric acid (HCl), tris(2-carboxyethyl)phosphine (TCEP), triethanolamine-buffer (TEOA; 0.3 M, pH 8) and 1-hydroxybenzotriazole (HOBt) were purchased from Aldrich (Milwaukee, WI). Dimethyl sulfoxide (DMSO), N-Acryloxysuccinimide (NAS) and, acetone ethanol were obtained from Fisher Scientific (Waltham, MA). Dialysis membranes (10000 MWCO, SpectraPor Biotech CE) were purchased from Spectrum Laboratories (Rancho Dominguez, CA). Paraformaldehyde (16% in H_2_O) was obtained from Electron Microscopy Sciences (Hartfield, PA). High molecular weight heparin (HMWH) was obtained from Santa Cruz Biotechnology, Inc (Dallas, Texas). The MMP-degradable crosslinker peptide (CQPQGLAKC), and bsp-RGD(15) adhesion peptide (CGGNGEPRGDTYRAY) were synthesized by United BioSystem Inc (Herndon, VA). All chemicals were used as received. All cell culture reagents and 1× Dulbecco's phosphate buffered saline (DPBS), rhodamine labelled phalloidin were purchased from Invitrogen (Carlsbad, CA).

### Synthesis of AcHyA hydrogel

HyA based hydrogels were synthesized using previously reported methods.[34] Briefly, HyA derivative carrying hydrazide groups (HyAADH) was synthesized using previously described methods[72], and acryloxysuccinimide (700 mg) was subsequently reacted with the HyAADH solution (300mg, 100 mL DI water) to generate acrylate groups on the HyA (AcHyA)[34–36]. Then, AcHyA-RGD derivative was synthesized by reacting CGGNGEPRGDTYRAY (bsp-RGD(15)) (10mg) with AcHyA solution (25mg, 10mL DI water) at room temperature. Separately, thiolated-heparin was synthesized by reacting heparin (50mg, 10mL DI water) with the excess of cysteamine in the presence of EDC and HOBt at pH 6.8. AcHyA (4mg), AcHyA-RGD (6 mg), and heparin-SH (0.03 wt%) were dissolved in 0.3 mL of TEOA buffer, then HyA hydrogels were fabricated by *in situ* crosslinking of the HyA precursors with bis-cysteine containing MMP-13 cleavable peptide (3mg, 50 μL TEOA buffer).

### Human CDC isolation and characterization

hCDC were generated from endomyocardial biopsies, as per previous reports.[41,73] Biopsies were obtained from patients by informed written consent, and all procedures were approved by the UCSF Intsitutional Review Board (10–01233). Routinely, hCDC were cultured on Fibronectin-coated plates in Iscove’s Modified Dulbecco’s Medium(IMDM) basal media containing 20% Fetal bovine serum (FBS), 1% L-Glutamine, 0.1 mmol/L 2-mercaptoethanol and 1% Penicillin-Streptomycin. Flow cytometry was used to characterize the hCDC, which were shown to express surface markers, CD31 (16%), CD34 (5%), CD90 (12%), CD105 (96%) and c-kit (5%). The hCDC were negative for CD45.

### Cell culture and encapsulation within hydrogel

Confluent hCDC were released using TrypLE and encapsulated in the hydrogels at the density of 5x10^6^ cells/mL as described in our previous report.[34] Three groups were assessed in this study: cells encapsulated in the presence of TGF-β1 (40 nM), cells encapsulated in the absence of TGF- β1, and cells encapsulated with TGF-β1 (40 nM) but pre-incubated with a CD105-blocking antibody prior to encapsulation. Cells were used from passage 2–7 in all experiments.

### Cell survival

Cell viability was assessed 24 hrs after encapsulation by a Live/Dead staining kit. Briefly, cell/hydrogel constructs were washed with PBS and incubated with Calcein/Propidium iodide, and visualized using a Prairie two photon/confocal microscope (Prairie Technologies, Middleton, WI).

### Cell spreading

Cell adhesion and spreading in the hydrogel was assessed at day 3. Samples were fixed using 4% (v/v) paraformaldehyde for 30 min and permeabilized with 0.1% Triton X-100 for 5 min. Following a PBS wash, cell/hydrogel constructs were incubated with F-actin stain (details), followed by a DAPI stain. Cell/hydrogel constructs were visualized using a Prairie two photon/confocal microscope (Prairie Technologies, Middleton, WI).

### Cell metabolic activity and proliferation

Metabolic activity was assessed at pre-determined timepoints by incubating the cell/hydrogel constructs with a 10% Alamar Blue (details) solution and analyzing according to the manufacturers instructions.

Cell proliferation was assessed at day 14 and compared with samples at day 1. At the appropriate timepoint, cell/hydrogel constructs were washed with PBS and treated with hyaluronidase (3000 U/ml). The released cells were analysed for cell proliferation using the CyQUANT cell proliferation assay (Thermo Fisher Scientific,).

### Immunocytochemistry

For immunocytochemistry, hydrogel samples were fixed using 4% (v/v) paraformaldehyde for 30 min and permeabilized with 0.1% Triton X-100 for 5 min. After blocking with 3% BSA for 1 hr, hydrogel samples were incubated overnight at 4°C with a 1:200 dilution of primary antibody (rabbit anti-CD31 IgG). After washing the cells with PBS, hydrogel samples were incubated with a 1:200 dilution of goat anti-rabbit AlexaFluor Texas red IgG (Invitrogen, Molecular Probes) for 2 hr at RT. Prior to imaging, cell nuclei were DAPI stained for 5 min at room temperature. Cell-gel constructs were visualized using a Prairie two photon/confocal microscope (Prairie Technologies, Middleton, WI).

### Analysis of VEGF_165_ production from encapsulated hCDC

Cell/hydrogel constructs were cultured in 600 μL cell culture media. At 14 days, the surrounding culture media and gels were collected and digested in hyaluronidase (3000 unit/mL). Subsequently, supernatants were collected after centrifugation (3000 rpm, 5 min) of the degraded hydrogels. The mass of MMPs and VEGF_165_ secreted by the entrained cells in collected supernatant was determined using sandwich ELISA kits (RayBiotech, Inc., Norcross GA).

### Analysis of NO production from encapsulated hCDC

At pre-determined timepoints, media was collected from the hCDC samples and analysed for its NO content via the Griess assay (Promega).

### Human angiogenesis protein profiler array

The expression of a range of angiogenesis-related proteins synthesized by encapsulated hCDC in each group was compared using an angiogenesis protein array (Proteome Profiler Human Angiogenesis Array Kit, R&D Systems, Minneapolis, MN), following the manufacturer’s instructions. The array membrane was visualized by a chemiluminiscence substrate under Bio-Rad ChemiDoc XRS System. The relative expression of the angiogenesis proteins produced by the CDCs in each of the hydrogels was measured by subtracting background and normalizing to positive controls, then comparing the pixel density of each chemiluminescence image.

The free online tool ClustVis was used to perform z-score transformations and to create heatmaps and dendrograms.[74]
